# Exercise increases interleukin-10 levels both intraarticularly and peri-synovially in patients with knee osteoarthritis: a randomized controlled trial

**DOI:** 10.1186/ar3064

**Published:** 2010-07-01

**Authors:** Ida C Helmark, Ulla R Mikkelsen, Jens Børglum, Anders Rothe, Marie CH Petersen, Ove Andersen, Henning Langberg, Michael Kjaer

**Affiliations:** 1 Institute of Sports Medicine, Department of Orthopaedic Surgery, Bispebjerg Hospital and Centre for Healthy Aging, Faculty of Health Sciences, University of Copenhagen, Bispebjerg Bakke 23, 2400 Copenhagen NV, Denmark; 2 Department of Anaesthesiology and Intensive Care Medicine, Bispebjerg Hospital, Bispebjerg Bakke 23, 2400 Copenhagen NV, Denmark; 3 Clinical Research Centre and Dept. of Infectious Diseases, Hvidovre Hospital, Kettegård Allé 30, 2650 Hvidovre, Denmark

## Abstract

**Introduction:**

The microdialysis method was applied to the human knee joint with osteoarthritis (OA) in order to reveal changes in biochemical markers of cartilage and inflammation, intraarticularly and in the synovium, in response to a single bout of mechanical joint loading.

**Methods:**

Thirty-one female subjects with OA of the knee were randomized to non-exercise (NEx) or exercise (Ex) groups. Following acute resistance exercise (25 sets of 10 repetitions at 60% of 1 Repetition Maximum) or none (NEx), peripheral nerve blocks just below the inguinal ligament were applied and two microdialysis catheters were positioned in two different compartments, intraarticularly and peri-synovially. The microdialysis catheters were perfused at a slow rate (2 μl/minute) with a solution of Ringer-acetate and radioactively labelled glucose allowing for determination of relative recovery (RR) and calculation of interstitial concentrations of inflammatory and cartilage biomarkers over a three-hour period.

**Results:**

A significant increase of Interleukin (IL) -10 was discovered in both positions of the knee in the Ex group over the three hours post exercise, whereas IL-10 remained stationary over time in the NEx group. IL-6 and IL-8 displayed significant increases over time regardless of group and position of the catheter. Cartilage oligomeric matrix protein (COMP) decreased intraarticularly in the post exercise period in the Ex group compared to the NEx group.

**Conclusions:**

Exercise caused an increase in both intraarticular and peri-synovial concentrations of IL-10 in a group of human females with knee OA. This suggests a positive effect of exercise on a chondroprotective anti-inflammatory cytokine response in patients with knee OA and might contribute to explaining the beneficial effect that exercise has on OA.

**Trial registration:**

NCT01090375.

## Introduction

Osteoarthritis (OA) is associated with cartilage erosion and bony changes as well as with intermittent periods of synovial membrane inflammation and subsequent release of biomarkers for inflammation [1]. Excessive loading of the joint in these patients can lead to a worsening of the pathology with an enhanced inflammatory response, joint pain and swelling [2]. On the other hand regular moderate exercise like strength training, cycling or walking is known to be advantageous in OA patients with no sign of deterioration of the inflammatory periods [3-5]. It is unknown how exercise exerts its beneficial role in OA, and whether it mainly is accomplished via a stabilisation of the joints through muscle strength and control, or whether exercise has a direct effect upon the joint cartilage and the synovium. Exercise has been proposed to positively modulate low-grade inflammation in elderly patients [6] and has been shown to have a positive effect on the glycosaminoglycan (GAG) content in cartilage of subjects at increased risk for OA [7]. It is therefore possible that acute exercise may induce changes in the intraarticular and peri-synovial milieu that encourages anti-inflammatory activity as well as releases potential chondroprotective substances, for example, Interleukin (IL)-10.

We have previously shown that microdialysis can be used as a method to investigate, continuously over time, the peri-synovial interstitial tissue and the joints space in parallel [8]. Several biomarkers for both cartilage (Aggrecan, COMP and CTX-II) and inflammation (Interleukins) have been studied in the circulating blood, the urine and intraarticularly [9-11]. In the present study the microdialysis method enabled us to investigate biochemical changes within and around the joint simultaneously in both the resting state and in a joint that had been subjected to exercise. The aim of the present study was, by applying the microdialysis technique, to monitor markers of cartilage breakdown and inflammation intraarticularly and in the synovium in a group of human females with knee OA over a period of three hours.

## Materials and methods

### Subjects

Thirty-one Caucasian women with symptomatic knee OA and fulfilling ACR (American College of Rheumatology) criteria participated after informed consent was obtained, in the study that was approved by the local Ethics Committee (H-KF-306126) and conducted in compliance with the Helsinki Declaration. Subjects were physically active and had no regular daily intake of pain medication or any other anti-inflammatory medication. None of the subjects had been exposed to any kind of surgery or major acknowledged trauma to the knee that was examined. Radiographs were taken in order to determine the Kellgren-Lawrence (K-L) grade and only subjects with K-L ≥ 1 were included. Subjects were randomized to either non exercise (NEx) (n = 13) or exercise (Ex) (n = 16). Two women were withdrawn from the study on the test day due to inadequate effect of the peripheral nerve blocks; hence, data are given for the 29 subjects who completed the study. Subjects were instructed to follow normal daily activities, but to refrain from strenuous exercise 24 hours prior to the test day. On the test day they were asked to travel by car or with public transportation to the hospital.

### Exercise protocol

A one-legged knee extension protocol was used. After a five-minute warm-up on a bicycle subjects were positioned in a leg-press machine (Techno Gym, Gambettola (FC), Italy) in an upright position with the knee bent in 90° and then performed a five to seven RM (Repetition Maximum) test. Hereafter, a working load of approximately 60% of one RM was applied and subjects completed 25 sets of 10 repetitions starting every one and a half minutes. During each set of 10 repetitions the working leg was extended against a vertical plate, while the resting leg was positioned on a horizontal plate. If the subjects experienced intolerable discomfort during the exercise, adjustment of the weight or seating position was attempted.

### Procedure

**Figure 1:**
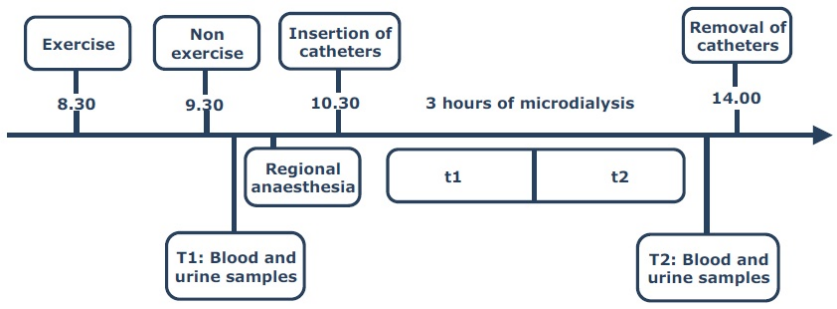
**Test day**. Subjects met at either 8.30 a.m. (Ex) or 9.30 a.m. (NEx) according to randomization. Following exercise or none, blood and urine samples were taken (T1) and regional anaesthesia was applied. Two catheters were hereafter positioned in the suprapatellar recess and in the sub synovial tissue on the medial side of the knee, respectively. Catheters were removed after three hours of microdialysis. The removal was preceded by blood and urine samples (T2). Samples of dialysate were collected every 30 minutes. Relative recovery was calculated for every sample and samples were later pooled (t1 = Sample 1 to 3; t2 = Sample 4 to 6).

**Figure 2:**
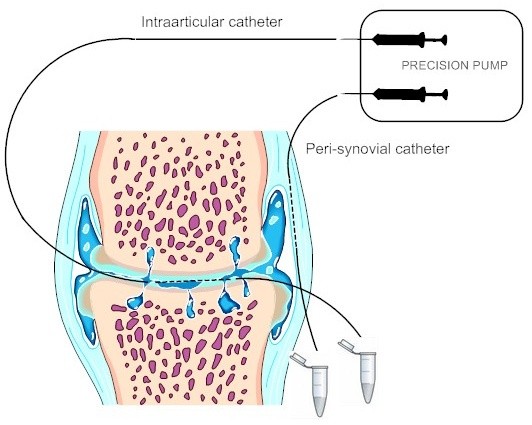
**Schematic illustration of the positioning of the catheters in the knee joint with osteoarthritis**. The intraarticular catheter is placed in the suprapatellar recess and the peri-synovial catheter in the medial part of the knee capsule. A precision pump perfuses the catheters at a selected rate of 2 μl/min.

On the test day subjects met at either 8.30 (Ex) or 9.30 (NEx) a.m. Subjects in the Ex group completed the exercise protocol and then proceeded with the same schedule as the NEx group (Figure 1). Blood and urine samples were taken after approximately 20 to 30 minutes of rest followed by the application of the ultrasound guided peripheral nerve blocks within one hour after exercise, that is, at the latest at 10.30 a.m. The ultrasound guided peripheral nerve blocks consisted of the so-called proximal triple block; that is, blocks of the femoral, obturator (anterior branch) and the lateral femoral cutaneous nerve. The blocks, applied by an anaesthesiologist, provided consistent effective anaesthesia of the knee for the time required for insertion of the catheters. The effect of the triple block gradually disappeared during the microdialysis period. The microdialysis catheters were positioned intraarticularly and close to the synovial tissue but extraarticularly, respectively (Figure 2 for schematic illustration). The positioning procedure has previously been tested in a pilot study on patients undergoing planned arthroscopy of the knee [8]. Both microdialysis catheters were connected to the microdialysis pump and perfused at a perfusion rate of 2 μl/minute. Samples were collected every 30 minutes and weighed before storage at -80°C.

### Principles of microdialysis

The custom-made microdialysis catheter consists of an inlet and an outlet tube connected by a 30 mm long membrane with a pore size of 3,000 kDa and a diameter of 0.4 mm [12]. The inlet tube is connected to a precision pump (2 μl/min) and hereby a state of near-equilibrium is reached around the membrane. The dialysate collected from the outlet tube contains molecules from the region of interest in which the catheter was positioned, making it possible to calculate the interstitial concentration of different molecules. In the present study we perfused the catheters with a Ringer solution containing radioactive labelled glucose (D-(3-^3^H)glucose) in order to determine the relative recovery (RR), a measure of the exchange rate of substances over the membrane. Relative recovery was calculated from the following equation:

Where P_dpm _and D_dpm _refers to the activities (disintegrations per minute) of the labelled glucose for the perfusate and the dialysate respectively. The interstitial concentrations are calculated using the internal reference calibration method equalling (C_d _- C_p_)/RR, where C_d _is dialysate concentration and C_p _is perfusate concentration [13].

### Samples

Blood and urine samples were collected in a standardized manner at the same time point in the morning (T1) and just before removal of the microdialysis catheters in the afternoon (T2). Blood samples were taken from the antecubital vein after a resting period of 20 to 30 minutes. Samples were centrifuged at 3,880 rpm and 4°C for 10 minutes and immediately after frozen at first -20°C and then -80°C until analysis. Microdialysis samples were collected every 30 minutes, weighed and 2 × 3 μl were taken from each sample in order to determine the activity (in duplicate) of the labelled glucose before samples were frozen at -80°C.

### Analysis

Samples were analyzed for COMP, Aggrecan, CTX-II, Interleukin (IL) -6, IL-8, IL-10 and Tumor Necrosis Factor (TNF)-α. Concentrations were determined with commercially available assays (COMP^® ^ELISA, AnaMar Medical AB, Sweden; Total Aggrecan for Culture^® ^ELISA; Urine and Serum Preclinical Cartilaps^® ^ELISA, IDS Nordic, Denmark; Milliplex^®^MAP, Millipore, Billerica, Massachusetts, USA). Samples were run in duplicate for all of the cartilage markers; inflammatory markers were run in duplicate when the amount of dialysate allowed it (approximately one third of the samples). COMP was determined in microdialysate and serum; Aggrecan in microdialysate, serum and urine; CTX-II in urine and inflammatory markers in microdialysate. Urinary creatinine was measured by a routine chemistry method and used for the calculation of creatinine-corrected urinary CTX-II and Aggrecan concentrations. Due to the small amount of microdialysate and low concentration of cartilage biomarkers it was not possible to perform analysis for every microdialysis sample, instead Sample 1 to 3 (denominated t1) and Sample 4 to 6 (denominated t2) were pooled and analyzed together.

### Statistics

Data on subjects' characteristics are given as mean ± SD and Students *t*-test was used to compare between groups. A one-way ANOVA was used to determine if there were differences in RR over the three hours of microdialysis. Results on biomarkers and cytokines are illustrated in figures with a before/after value and a connecting line. Comparison was done using non-parametric statistics due to the non normal distribution of data. Wilcoxon matched pair test for comparison within a group, and Mann Whitney test for comparison between groups. Level of significance was set at a two-tailed *P*-value of 0.05.

## Results

### Subjects

Twenty-nine subjects completed the test day, the Ex group with an average work load of 61 ± 7% of oneRM (mean ± SD). No differences were found between the non-exercise (NEx) and exercise (Ex) groups with regards to age, BMI, K-L grade or pain (Table 1). The KOOS questionnaire, which was filled in on the test day, did not show any differences between the two groups in any of the measured parameters (Pain, Symptom, Function in daily living, Function in sport and recreation and knee related Quality of life). Subjects completed the exercise protocol without reporting any unusual discomfort (pain or swelling) of the knee during the exercise or in the hours/days following the exercise. (A telephone interview was conducted two days after the test day).

**Table 1:** Demographics of the subjects

	Age (years)	BMI (kg/m^2^)	K-L grade	Pain
**NEx (N = 13)**	67 ± 7	25.1 ± 2.6	2.5 ± 0.8	69 ± 16
**Ex (N = 16)**	66 ± 6	26.4 ± 2.8	2.3 ± 0.9	64 ± 13

Data are presented as mean ± SD, no significant differences were found between groups. Pain was registered using the KOOS questionnaire with 100 indicating no symptoms and 0 indicating extreme symptoms. (BMI: Body Mass Index, K-L: Kellgren-Lawrence).

### Method

Relative recovery was calculated for each of the samples recovered from the 29 subjects; however, in five subjects no peri-synovial catheters were inserted due to technical difficulties from relative larger volumes of adipose tissue in some subjects. The average RR from the intraarticular and peri-synovial catheters was unaffected by exercise, that is, there was no difference between the NEx and Ex groups, but in the NEx group alone there was a significant difference in RR between the two compartments (*P *< 0.05, results not shown). No difference in RR was found over the course of time, that is, from Sample 1 to Sample 6, overall or in NEx and Ex groups respectively (one-way ANOVA, *P *> 0.05).

### Inflammatory markers

**Figure 3:**
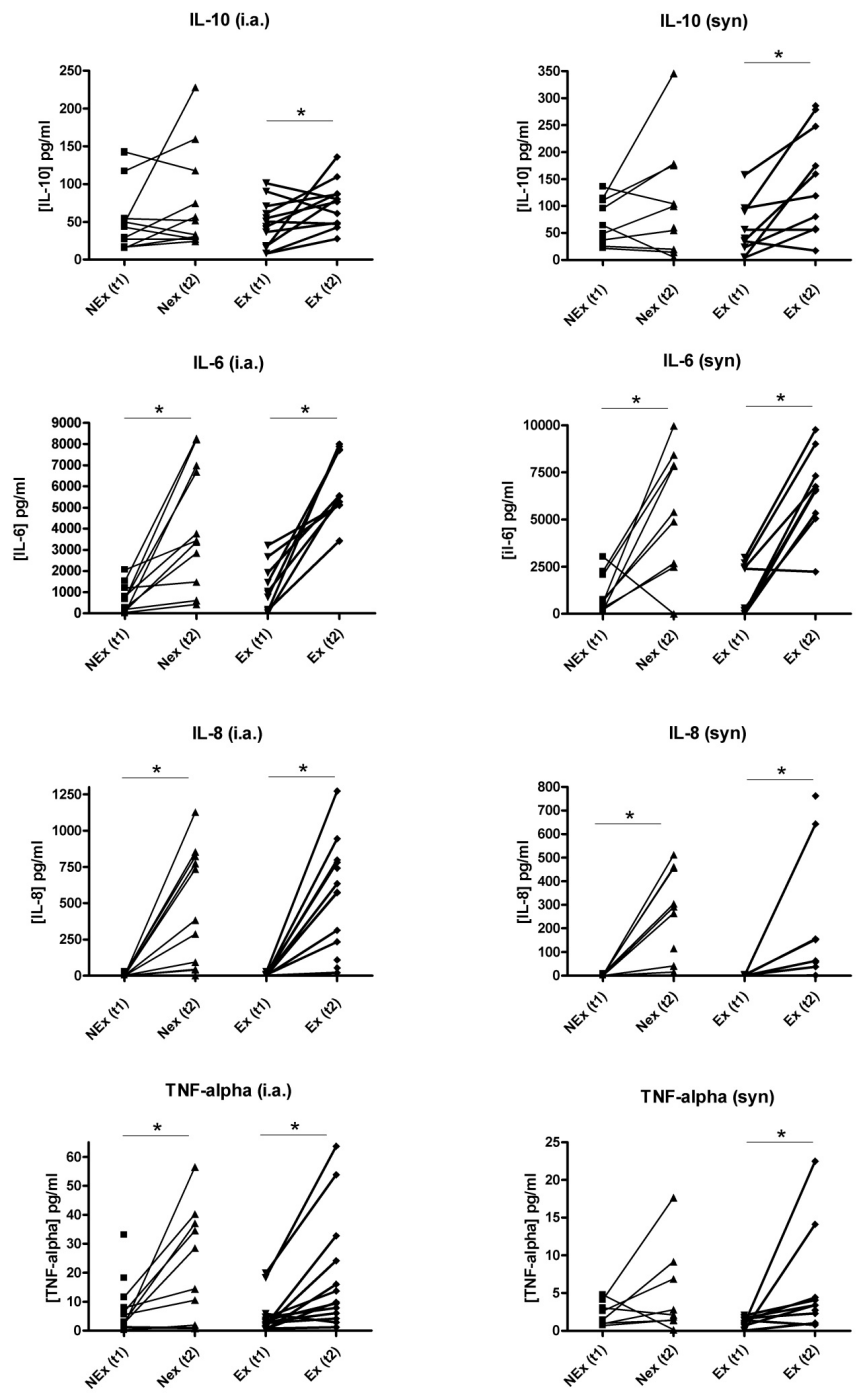
**Concentrations of IL-6, IL-8, IL-10 and TNF-α**. Intraarticular (left panel) and peri-synovial (right panel) concentrations in the NEx and Ex groups at T1 and T2. Each point represents a mean (when run in duplicate) of the measured values and each connecting line represents a subject with before/after values. IL-10 increased significantly in the Ex group in both compartments (*P < 0.05), but remained stationary in the NEx group. Significant increases regardless of exercise are seen for IL-6 and IL-8 in both positions and also for TNF-α intraarticularly (*P < 0.05), whereas TNF-α increased peri-synovially in the Ex group but not in the Ex group. (i.a., intraarticular; syn, peri-synovial).

The IL-10 concentration showed significant increases in both the peri-synovial and the intraarticular compartments (*P *< 0.05) (Figure 3). The rise in IL-10 concentrations was found only in the Ex group whereas the NEx group maintained a stationary level. A highly significant increase was found over time for IL-6 and IL-8 in both positions regardless of exercise. Intraarticular TNF-α levels were significantly elevated in both groups, but for the peri-synovial levels an increase was found only in the Ex group. Due to levels below the minimum detectable concentration are only few results available for IL-8 concentrations peri-synovially. No differences were found between groups at t1 for either of the cytokines.

### Cartilage markers

**Figure 4:**
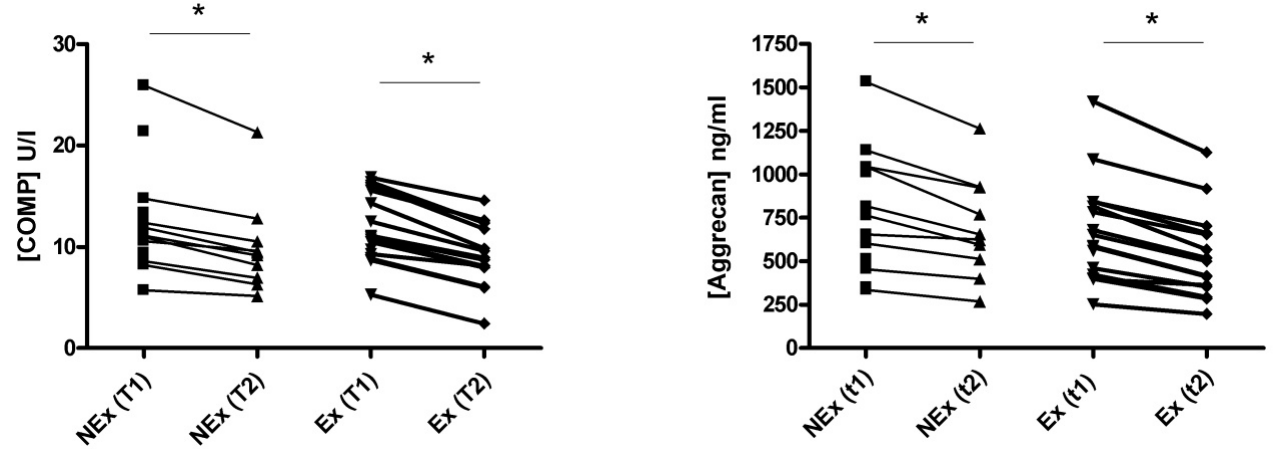
**Serum concentrations of COMP and Aggrecan in the NEx and Ex groups at T1 and T2**. Each point represents a mean of the measured values and each connecting line represents a subject with before/after values. A significant decrease over time was found in both groups for both markers, regardless of exercise (*P < 0.05).

**Figure 5:**
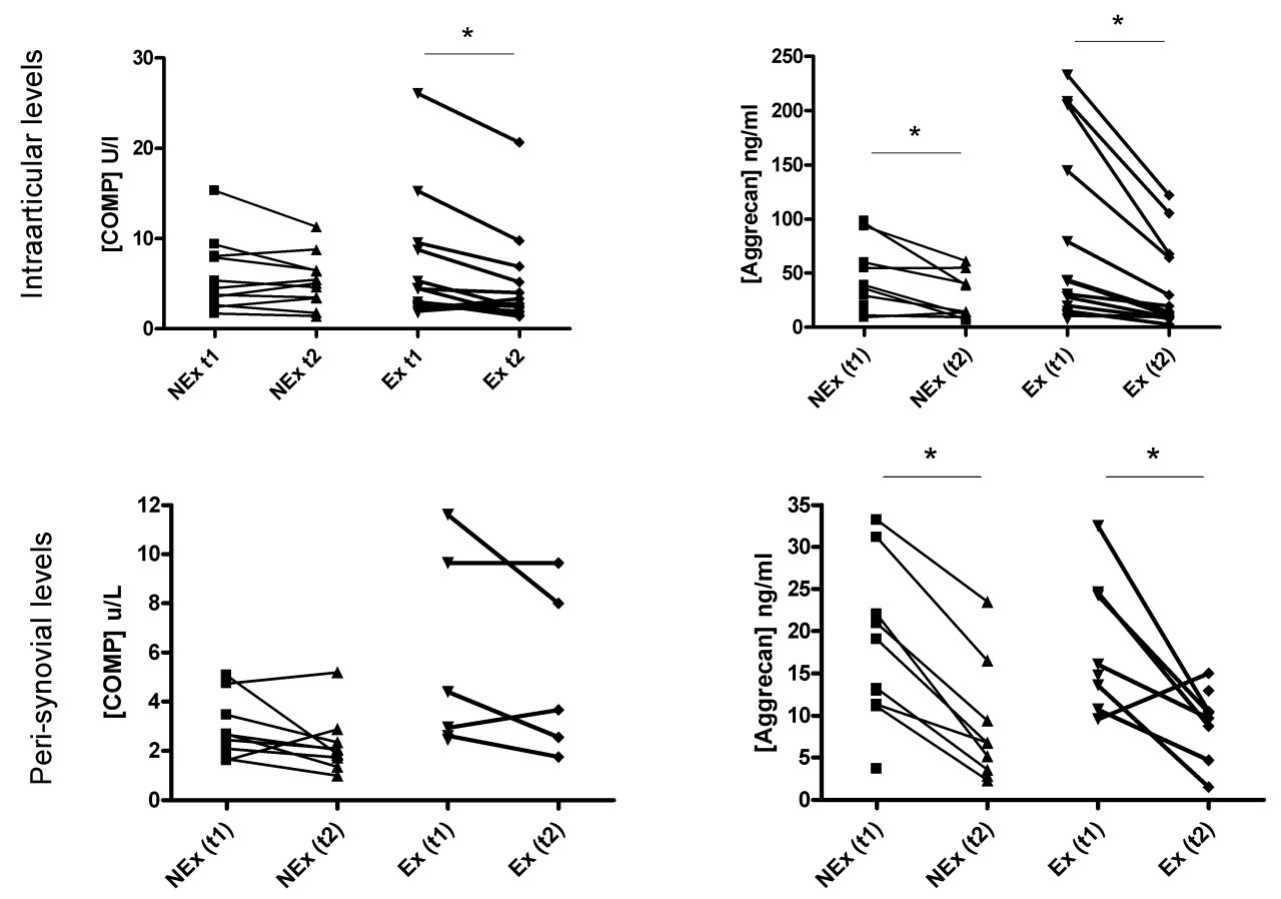
**Intraarticular (top row) and peri-synovial (bottom row) concentrations of COMP and Aggrecan**. Each point represents a mean of the measured values and each connecting line represents a subject with before/after values. COMP remained stationary in the NEx group in both compartments and in the Ex group peri-synovially over time, but decreased in the Ex group intraarticularly, whereas Aggrecan concentrations decreased significantly from T1 to T2 in both positions regardless of exercise (*P < 0.05).

**Figure 6:**
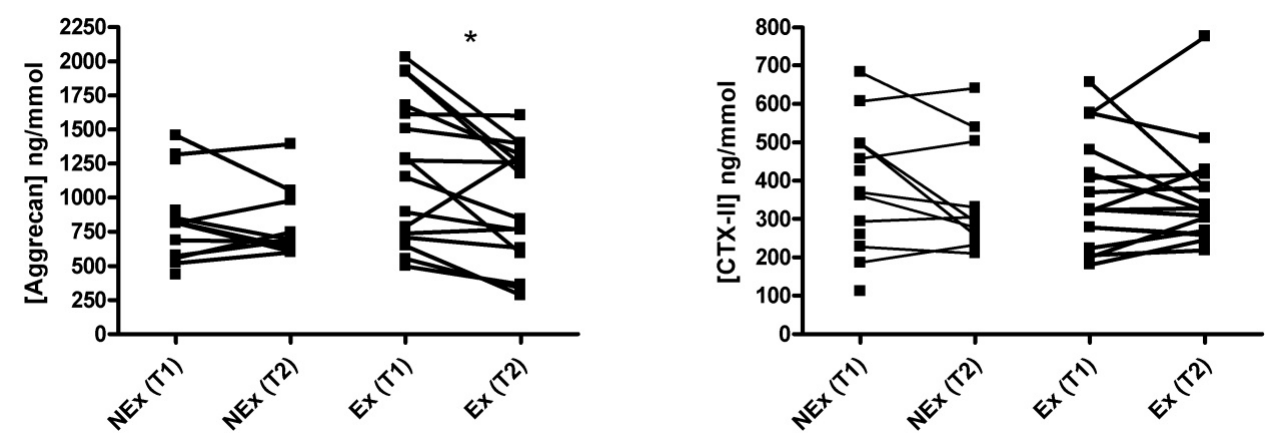
**Creatinine-corrected urinary concentrations of Aggrecan and CTX-II in NEx and Ex groups at T1 and T2**. Each point represents a mean of the measured values and each connecting line represents a subject with before/after values. The Ex group had a significantly higher level of Aggrecan in the urine immediately after exercise compared to the values after three hours of microdialysis (rest), whereas the NEx group showed no significant difference between T1 and T2 (*P < 0.05). CTX-II displayed no changes over time.

Serum measurements showed a significant decrease in COMP and Aggrecan concentrations over time in both groups, but no difference was found between groups at T1, where the Ex group had already completed the exercise protocol (Figure 4). The intraarticular microdialysate concentration of COMP decreased in the Ex group from t1 to t2, whereas the level in the NEx group was stationary from t1 to t2 (Figure 5). Aggrecan concentration decreased in both groups from t1 to t2 intraarticularly. No difference was found between groups at t1 for neither COMP nor Aggrecan (Figure 5). Aggrecan did similarly decrease significantly in both groups over time in the peri-synovial compartment, whereas COMP did not change from t1 to t2 in either group (Figure 5). Due to values below the detection limit only five sets of paired values were obtained in the Ex group. The urinary concentration of Aggrecan was significantly elevated in the Ex group at T1 compared to T2. After three hours of microdialysis the level returned to the level of the NEx group (Figure 6).

## Discussion

The microdialysis method was applied to a group of human females with OA of the knee in order to obtain information about the effect of a single bout of mechanical loading on cartilage biomarkers and cytokines both inside the joint as well as in the synovium over a period of three hours. The present study demonstrates that the anti-inflammatory cytokine, IL-10, increased significantly over time post exercise in the Ex group in both compartments, but not in the NEx group, indicating that the increase observed in the Ex group could be ascribed to exercise (Figure 3). Most previous studies on IL-10 and OA have been conducted on animals or *ex vivo*, and only a few investigators have also measured the concentration of IL-10 in synovial fluid in humans with OA, revealing concentrations in accordance with the present findings [14,15] or in some cases showing non detectable concentrations [16,17]. To our knowledge, the intraarticular and the peri-synovial concentration responses to exercise have never been investigated in OA previously. It is of interest that IL-10 responds positively to mechanical joint loading in this patient group, as IL-10 has been found to display chondroprotective properties by antagonizing important steps in the suggested pathogenesis of OA, such as suppressing the release of inflammatory mediators by macrophages and the activation of synoviocytes and chondrocytes [18,19]. The previously mentioned study by Fraser *et al *[14] found that patients with early psoriatic arthritis and early rheumatoid arthritis have higher levels of IL-10 compared to patients in later stages of the disease. It appears that development of the disease leads to an impaired capacity to suppress the release of inflammatory mediators and thus a decreased immunoresponse beyond a certain point in the disease course. Taking into account the present data, this would argue in favour of stimulating the joints of patients with early OA, or patients at high risk of developing OA, with exercise, as this would result in a release of IL-10, a response that potentially could have beneficial effects upon the control of the immune response. The general influence of exercise on IL-10 has not been investigated formerly in human joint aspirates of synovial fluid and previous studies on serum levels show some inconsistency [20-23]. One study has, in support of our results, demonstrated that higher levels of regular physical activity are associated with increased levels of IL-10 in the blood of healthy older males, whereas studies on young, moderately and well-trained males in different training sessions have shown both increases as well as a decrease in serum IL-10 concentrations. Increased production of anti-inflammatory cytokines during exercise can possibly restrict the production of pro-inflammatory cytokines such as IL-6, IL-8 and TNF-α. *In vitro *studies suggest that IL-6 act as a negative regulator of chondrocyte proliferation and articular cartilage metabolism [24] and that IL-8 possibly act as a modulator of both IL-6 and TNF-α as well as a chemotactic agent for neutrophils [25]. Hence, these cytokines play an important role in the low-inflammatory response of OA. In our study we determined highly significant increases in IL-6 and IL-8 concentrations from the first hour and a half of sampling to the second hour and a half of sampling; regardless of exercise and position of the catheter (Figure 3). Our levels reached medians of approximately 600 pg/ml and 5,000 pg/ml for each of the sampling periods irrespective of the position of the catheter, which contrasts with the findings of other investigators, who discovered levels of IL-6 and IL-8 in synovial fluid obtained by direct joint puncture of approximately 50 to 200 pg/ml [11,26,27]. A major trauma to the knee such as a tear of the anterior cruciate ligament has been found to result in IL-6 levels above 20,000 pg/ml within the first couple of days [28], and as the concentrations in the present study increased over time our findings could indicate that the insertion of the catheters *per se *induced a production of these cytokines due to the tissue injury. TNF-α concentrations show a pattern similar to IL-6 and IL-8, but for the peri-synovial level an increase was found only in the Ex group. The overall intraarticular concentrations of TNF-α at t1 in our study are somewhat below what has previously been reported [14,27,29]. We have no obvious explanation for this finding, but it could be speculated that the known suppressive effect of IL-10 on TNF-α [18,30] could have contributed to the present findings.

Many biomarkers of cartilage measured in blood are known to present a circadian variation with concentrations being lower during the night and higher during daytime [9,31], most likely due to an effect of the tissue resting. In the present study we have confirmed this *resting effect *as serum concentrations of COMP and Aggrecan decrease over the course of the test day (Figure 4). Other studies have detected a temporary increase in serum COMP following exercise [32,33], which was not the case in our study, maybe due to the exercise protocol not being strenuous enough to induce systemic changes. We have, however, demonstrated for the first time that unloading of the joints for three hours results in an immediately measurable decrease in Aggrecan inside a single joint as well as in the synovium (Figure 5) despite previous physical activity. This is in agreement with the suggestion that Aggrecan is one of the first fragments to be released during cartilage breakdown. A high metabolism of Aggrecan would lead to an increased excretion through the urine, and it could then be expected that a higher urinary concentration would be seen in the Ex group, reflecting a faster turnover. The difference between the two groups at that point (T1, where the Ex group had already performed the exercise protocol as compared to the NEx group) did, however, not reach statistical significance. Instead a significant difference was found within the Ex group, indicating a faster reversion to baseline level (Figure 6). The concentration of CTX-II, a degradation product from collagen II, was not affected by a single bout of exercise in our subjects, and it is likely that the systemic measurement was too crude and insensitive to be able to detect any contribution from a single joint. It probably requires long term adaptation to loading to change the CTX-II level which has been demonstrated by O'Kane *et al*. in elite athletes performing different kinds of sports [34] and in OA patients followed for years [35]. Interstitial concentration of COMP did not reveal any changes in the NEx group in either of the compartments, but in the Ex group we found a significant decrease intraarticularly but not peri-synovially (Figure 5). A plausible reason for the intraarticular decrease could be the increased blood-flow and hydrostatic pressure following exercise, which could lead to a faster elimination of the COMP molecules in the Ex group. The turnover of cartilage is very slow with an estimated half life of collagen II of more than 100 years and for Aggrecan of 3 to 24 years [36,37]. It is therefore not realistic that a single bout of exercise should have caused a molecular rearrangement of the cartilage; hence, the measured molecules must have been present in the joint space or very close to the cartilage surface during the exercise.

The microdialysis method used in the present study has been applied to a variety of human tissues including brain, adipose tissue and peritendinous tissue [38-40] and is generally considered a minimally invasive procedure compared to other tissue sampling techniques. It must be acknowledged, though, that the microdialysis method still causes tissue injury during the insertion of the catheters which by itself can generate an inflammatory response as shown by Langberg *et al *in peritendinous tissue and by Clough *et al*. [41] in relation to skin wounding and allergen-induced inflammation. A sharp rise in IL-6 and IL-8 concentrations was found in the area with inflammation, although not to the same extent for IL-6 as in the present study. Importantly, Clough *et al *[41] also found an increase at a 1 cm distance from the insertion site, which indicates that the increased production of cytokines found in our study may be a result of a larger involvement of the already inflamed tissue next to the insertion site. Another important issue regarding the microdialysis method is the choice of tracer for determination of relative recovery. We used radioactively labelled glucose, which is a small molecule of only 0.18 kDa compared to Aggrecan and COMP (exceeding 500 kDa), and the chosen cytokines with a molecular weight of 11.1 to 25.6 kDa. This probably leads to an underestimation of the true concentrations (due to an overestimated RR) with regards to the cartilage markers. In addition, larger molecules such as markers of cartilage turn-over, do not move readily, which creates a possible risk of *drainage *from the area around the membrane. This must be considered since the concentrations of Aggrecan and COMP in our study are far from what joint puncture has shown in other studies on similar patients [42-44]. However, even taking these limitations into consideration, it is important to note that procedures were identical for Ex and NEx groups, and this should not influence the detection of potential differences between groups.

Because of the specific use of anaesthetic method in this study and due to the risk of infection it was not considered pertinent to have catheters inserted before and again after exercise. Even with a block of only sensory nerves the risk of damage to the catheters during exercise would be too high, so we were constrained to look at two different groups and compare these. We believe the two groups are very homogenous as the KOOS score and other demographics showed no differences between groups. The presented results are valid for at selected group of female subjects, all with a relatively low Body Mass Index compared to other OA patients (because the insertion of the peri-synovial catheter would otherwise be complicated by penetrating too much adipose tissue) and with no history of other inflammatory diseases or regular intake of anti-inflammatory drugs.

With the limited conservative treatment options for OA patients it is of great importance to uncover the benefits of different kinds of exercise in order to provide a possibility of self-management. It could be speculated if specific groups of knee OA patients with no contraindications should be encouraged to perform more loadbearing activities to further improve the effect of exercise.

## Conclusions

The present study demonstrates that it is possible to evaluate the effect of a single bout of exercise on cartilage and inflammatory markers in a group of female subjects with OA of the knee, over time and simultaneously in two different compartments of the knee, using the microdialysis method. We discovered a significant increase of the chondroprotective IL-10 only in the group that performed exercise compared to the non-exercising group intraarticularly as well as peri-synovially. This might contribute to explain the beneficial effect that exercise has on patients with knee OA.

## Abbreviations

ACR: American College of Rheumatology; COMP: Cartilage Oligomeric Matrix Protein; CTX-II: Telopeptide; Ex: Exercise group; GAG: glycosaminoglycan; IL: Interleukin; K-L: Kellgren-Lawrence; NEx: Non Exercise group; OA: Osteoarthritis; RM: Repetition Maximum; RR: Relative Recovery; TNF-α: Tumor Necrosis Factor α.

## Competing interests

The authors declare that they have no competing interests.

## Authors' contributions

ICH designed the study, led and performed the experimental procedure during test days, gathered and processed the data, and drafted the manuscript. URM participated in the study design and coordination and helped to draft the manuscript. JB and AR provided the anaesthesiological assistance. MCHP performed the immunoassays of the cartilage markers. OA contributed with the analysis of cytokines. HL and MK participated in the design of the study and participated in its design and coordination, and helped to draft the manuscript. All authors have read and approved the final manuscript.
